# Physicochemical Properties, Biological Activity, Health Benefits, and General Limitations of Aged Black Garlic: A Review

**DOI:** 10.3390/molecules22060919

**Published:** 2017-06-01

**Authors:** Ji Hyeon Ryu, Dawon Kang

**Affiliations:** 1 Department of Convergence Medical Science, Gyeongsang National University, Jinju 52727, Korea; wlgus9217@naver.com; 2 Department of Physiology and Institute of Health Sciences, College of Medicine, Gyeongsang National University, Jinju 52727, Korea

**Keywords:** aged black garlic, allicin, antioxidants, black garlic, pyruvate, sugar

## Abstract

Garlic (*Allium sativum*) has been used as a medicinal food since ancient times. However, some people are reluctant to ingest raw garlic due to its unpleasant odor and taste. Therefore, many types of garlic preparations have been developed to reduce these attributes without losing biological functions. Aged black garlic (ABG) is a garlic preparation with a sweet and sour taste and no strong odor. It has recently been introduced to Asian markets as a functional food. Extensive in vitro and in vivo studies have demonstrated that ABG has a variety of biological functions such as antioxidant, anti-inflammatory, anti-cancer, anti-obesity, anti-diabetic, anti-allergic, cardioprotective, and hepatoprotective effects. Recent studies have compared the biological activity and function of ABG to those of raw garlic. ABG shows lower anti-inflammatory, anti-coagulation, immunomodulatory, and anti-allergic effects compared to raw garlic. This paper reviews the physicochemical properties, biological activity, health benefits, adverse effects, and general limitations of ABG.

## 1. Introduction

Garlic (*Allium sativum*) has been taken as a folk medicine all over the world for the prevention and treatment of various diseases from ancient times through to the present day. Anciently, many cultures, including Babylonians, Chinese, Egyptians, Greeks, Hindus, Phoenicians, Romans, and Vikings, frequently ingested garlic as a folk medicine to treat flatulence, intestinal disorders, respiratory infections, skin diseases, wounds, and many other ailments [1]. Today, natural products and alternative medicines are popular amongst people concerned about potential adverse effects of conventional medicines. Garlic is a popular natural medicine, and recent studies have studied the function of garlic as a medicinal food. A wide array of therapeutic effects of garlic has been observed including anti-cancer, antibacterial, antiviral, anti-diabetic, anti-hypertensive, cardioprotective, hepatoprotective, hypolipidemic, and antioxidant effects, as well as immune enhancement. These effects are attributed to a high concentration of organosulfur compounds (OSCs) [2,3,4,5,6].

Despite the numerous health benefits of garlic, with the exception of China and India, global consumption of garlic is declining. Some people are reluctant to eat raw garlic because of its pungent taste and smell [7], and raw garlic can cause gastrointestinal discomfort in some people. Food scientists have developed aged garlic preparations to reduce these discomforts.

Aged black garlic (ABG), an aged garlic preparation, is known as a functional food and is popular in Asia. ABG is produced by application of high temperature and humidity over 10 days [8,9,10,11,12,13,14,15,16,17,18,19,20,21]. During the aging process, the odorous, harsh, and irritating compounds in fresh raw garlic (FRG) are converted naturally into stable and safe compounds [22]. As a result, ABG has a sweet and sour taste and jelly-like texture. The heating process leads to a Maillard reaction, creating the typical dark brown color, and produces antioxidant compounds [23,24,25]. ABG contains bioactive compounds, such as phenols, flavonoids, pyruvate, thiosulfate, S-allylcysteine (SAC), and S-allylmercaptocysteine (SAMC) [26,27,28,29,30,31]. In aged garlic extracts (AGE), SAC and SAMC are the major unique water soluble OSCs. Diallyl sulfide (DAS), diallyl disulfide (DADS), diallyl trisulfide (DATS), and diallyl tetrasulfide are oil soluble compounds in AGE. These OSCs, derived from allicin, are responsible for the antioxidant activity of AGE [22,32]. Compared to aged garlic, a small amount of literature regarding ABG has been published. The concentrations of oil soluble DAS, DADS, DATS, and diallyl tetrasulfide have not yet been analyzed in ABG.

In this review, we summarize the current knowledge regarding biological functions and physicochemical properties of ABG, its adverse effects, and general limitations. This review will provide direction to understand the health benefits of ABG, and to stress the importance of research about ABG’s component analysis.

During the preparation of this review we became aware of a very similar review on the properties and applications of ABG by Kimura et al. [33]. We recommend that our review be read in conjunction with theirs.

## 2. Preparation of ABG

### Manufacturing Protocol

**Figure 1:**
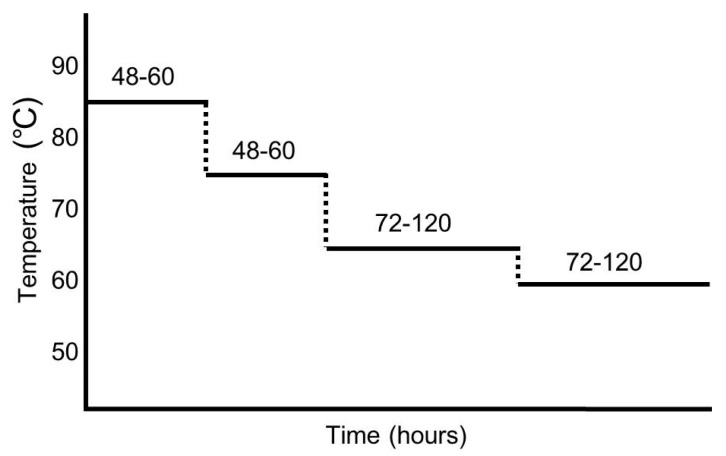
An example representing aging processes for aged black garlic (ABG) production. This protocol is from a patent (No. 10-2012-0058110). For ABG preparation, fresh raw garlic (FRG) was incubated for 48–60 h at 80–90 °C followed by 48–60 h at 70–80 °C, then 72–120 h at 60–70 °C, and finally 72–120 h at 55–65 °C.

Specific manufacturing protocols of ABG vary slightly according to manufacturers. ABG is manufactured from FRG in a temperature and humidity controlled room for over 10 days without any additional treatment or additives. The processing period for ABG could be changed according to the temperature, with shorter periods at higher temperatures [10]. ABG is generally prepared at the temperature range from 40–90 °C and a relative humidity (RH) of 60–90% [11,12,13,25,33,34,35]. Different temperature and incubation periods make a difference in the concentration of active components in garlic [35]. Generally, ABG is produced after multi-step heat treatment processes (see Figure 1). Most literature refers to garlic aged over 10 days, with multi-step heat treatment processes, as ABG. After the aging process, for the analysis of constituents, garlic compounds are extracted using suitable solvents.

Shin et al. [36] produced ABG in a four-step process similar to Figure 1. Step 1: Heating for 2 days at 90 °C. Step 2: Heating for 4 days at 80 °C. Step 3: Heating for 4 days at 60 °C. Step 4: Heating for 1 day at 40 °C. Bae et al. [34] produced ABG at 40, 55, 70, and 85 °C with 70% RH for 1, 3, 5, 10, 15, 30, and 45 days. Choi et al. [37] produced ABG at 70 °C in 90% RH for 7, 14, 21, 28, and 35 days. Kang [25] used a programmed stepwise schedule as follows: Step 1, 90 °C and 100% RH for 34 h; Step 2, 60 °C and 60% RH for 6 h; Step 3, 75 °C and 70% RH for 48 h; Step 4, 70 °C and 60% RH for 60 h; and Step 5, 65 °C and 50% RH for 192 h. The properties of ABG after the last processing step, or produced under high temperature and the longest aging period, are compared to those of FRG (Table 1, Table 2 and Table 3) [25,34,37]. Literature not mentioning FRG [36] was excluded in the comparison between ABG and FRG. The properties of ABG produced at each step are different, and the physicochemical properties are increased or decreased stepwise.

## 3. Properties of ABG

### 3.1. Physicochemical Properties

**Figure 2:**
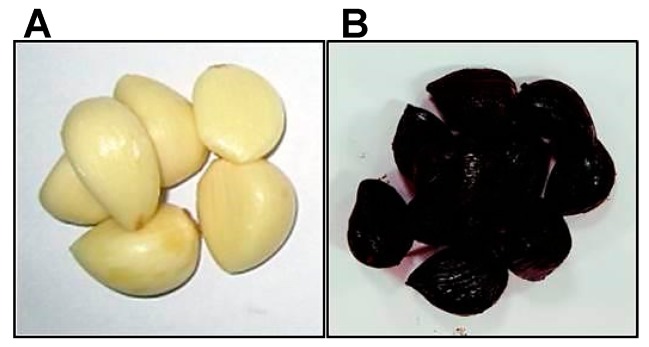
The colors of FRG and ABG cloves. The pure white/light yellow color of FRG (**A**) was changed to black (**B**) by heat treatment. The heating procedure was performed in a temperature and humidity controlled room for over 10 days (see Figure 1).

Color is an important physicochemical property that affects consumers’ perception of food. The pure white/light yellow color of FRG is changed to dark brown and eventually black during its aging process (Figure 2). Contents of moisture, protein, lipid, carbohydrate, and ash in garlics are determined according to the Association of Official Analytical Chemists (AOAC) method. There were commonalities in analytical methods among studies, and the methods used in each study are illustrated in Table 1, Table 2 and Table 3, except studies not mentioning analytical methods. Data found within studies are statistically significant (Table 1, Table 2 and Table 3). The concentrations of total and reducing sugars were detected by colorimetric method using phenol-sulfuric acid (PSA) and 3,5-dinitrosalicylic acid (DNS), respectively. ABG shows low moisture content and pH and high browning intensity compared to FRG. In addition, the contents of protein, lipid, carbohydrate, ash, total sugar, and reducing sugar are high in ABG (Table 1). The total and reducing sugar contents from both FRG and ABG show a significant difference throughout the literature. These differences could result from using different cultivars of garlic. Overall, the total and reducing sugar contents are higher in ABG than those in FRG.

Table 2 shows the changes in phytochemical components in ABG compared to FRG. Allicin and SAC contents were determined by high performance liquid chromatography (HPLC). Flavonoid, pyruvate, thiosulfate, and total phenol contents were determined by colorimetric methods. ABG is abundant in flavonoids, pyruvate, and phenols, but less abundant in allicin compared to FRG. The marked variation in concentrations of flavonoids and total phenols throughout the literature results from different bases used for calculation, such as quercetin, rutin, caffeic acid, garlic acid, and tannic acid. Changes in thiosulfate contents in ABG are controversial among researchers. The amount of SAC increased during ABG process is four- to eight-fold higher than that in FRG [14,15,34]. The amount of SAC is affected by the aging period rather than by temperature. The SAC contents in garlic aged at 40 °C and 85 °C for 24 h are 4.31 ± 0.01 and 2.88 ± 0.16 mg/100 g dry weight, respectively. However, the contents markedly increase to 12.47 ± 0.16 and 8.55 ± 0.08 mg/100 g dry weight in garlic aged at 40 °C and 85 °C, respectively for 45 days. Interestingly, the contents of SAC highly increase over time, but the contents decrease as temperature increases [34]. There are also variations in the detection levels of the SAC contents in ABG based on each method [15] (see Table 2). Generally, HPLC represents HPLC-ultraviolet detection (UVD). Some studies used HPLC-fluorescence detection (FLD) method. In a comparative study of the different analytical methods for analysis of SAC in garlic [15], HPLC-FLD and HPLC-UVD showed 2.2 and 2.3 mg/100 g SAC content in FRG, respectively. However, the SAC contents greatly vary in ABG depending on the method. The contents of SAC in ABG detected by HPLC-FLD and HPLC-UVD methods are 9.8 ± 0.2 mg/100 g and 11.4 ± 0.9 mg/100 g, respectively. Despite variation in the data due to analytical methods, SAC content in ABG is still higher than that in FRG. In addition, DPPH radical scavenging ability is increased by a high temperature and a long aging time [34]. Choi et al. [37] report that antioxidant contents in ABG, such as total polyphenol and total flavonoid, and antioxidant activities are high in garlic aged for 21 days compared to 35 days.

The concentrations of free sugars and minerals in garlic are shown in Table 3. The concentrations of free sugars and minerals increase in ABG. The concentrations were measured by HPLC with appropriate pretreatment. The high concentration of free sugars, in particular fructose, is closely related to the sweetness of ABG [31]. The rate of change in fructose concentration is very high compared to other free sugars, such as arabinose, galactose, glucose, sucrose, and maltose. ABG shows a trend of increase in concentration of amino acids, such as aspartic acid, threonine, serine, glutamic acid, proline, glycine, alanine, methionine, isoleucine, leucine, tyrosine, and phenylalanine [14,25,31]. Free amino acids are detected in ABG, but their concentrations have not yet been compared between ABG and FRG.

### 3.2. Antioxidant Activity, Compounds, and Therapeutic Effects

Antioxidant activity is the strongest property of ABG. The antioxidant and anti-inflammatory activities are generally determined by colorimetric methods with different substrates. Ferrous (Fe^2+^)-chelating ability, ferricyanide reducing power, free radical and nitrite scavenging activity, and superoxide dismutase (SOD) activity of ABG were analyzed and compared to those of FRG [9,11,36,39,40,41]. Comparative studies on biological activities between FRG and ABG show that ABG has strong DPPH, ABTS, hydroxy radical scavenging activities, reducing power, and SOD activity compared to FRG [9,14,16,36,38,39]. However, nitrite radical scavenging activity of FRG and ABG is controversial between two published studies [16,38]. One of two controversial studies used water extract of garlic for comparison of nitrite radical scavenging activity of FRG and ABG [38], and the other study used ethanol extract [16]. Water extract of ABG shows high nitrite radical scavenging activity compared to FRG, but ethanol extract of ABG shows the opposite. The ethanol extract of garlic increases nitrite radical scavenging activity in a dose-dependent manner. However, the scavenging activities vary according to concentration. The nitrite radical scavenging activity is detected low at concentrations below 10 mg/mL of ABG compared to FRG, but high at concentrations above 20 mg/mL. ABG shows low Fe^2+^-chelating activity compared to FRG (see Table 4). The number of studies regarding nitrite radical scavenging and Fe^2+^-chelating activities is small. The antioxidant activity of ABG is comparable to *N*-acetyl-l-cysteine (NAC), a representative ROS scavenger. These antioxidant activities result from various phytochemicals in ABG, giving several therapeutic advantages.

Representative antioxidant components in ABG are total phenols and flavonoids [31,36,41]. OSCs, such as SAC, SAMC, DAS, DADS, and DATS, are also important antioxidants in AGE [22,32,42], but there is no report about their presence and concentration in ABG, an aged garlic. Additional studies are needed to identify these components in ABG. ABG contains many kinds of antioxidants, such as tetrahydro-β-carboline derivatives [43,44], *N*-fructosyl glutamate, *N*-fructosyl arginine [45], allixin, selenium [5], and *N*-alpha-(1-deoxy-d-fructos-1-yl)-l-arginine (Fru-Arg) [46]. Pyruvate is the main antioxidant molecule [47,48], which is abundant in ABG [9,31,36]. The concentration of pyruvate in ABG is higher than that in FRG (see Table 2). Pyruvate reduces H_2_O_2_-induced ROS levels in RAW264.7 cells [9].

ABG inhibits H_2_O_2_-induced ROS generation in RAW264.7 cells [9,41] and *tert*-butyl hydroperoxide-induced lipid peroxidation in isolated rat hepatocytes [49]. ABG also shows antioxidant activity in animal models. Ultraviolet B-induced oxidative damage in mice skin is reduced by ABG treatment [50]. ABG reduces chronic alcohol-induced oxidative liver damage [51] and hangover symptoms [52] in rats. Lipid peroxidation is downregulated by ABG in high-fat-diet (HFD) rats [17,53,54] and restraint stressed rats [55].

### 3.3. Anti-Inflammatory Activity, Compounds and Therapeutic Effects

Compared to studies regarding antioxidant activity of ABG, a relatively small number of studies have considered the anti-inflammatory activity of ABG. Some compounds showing anti-inflammatory effects in ABG are identified as pyruvate, 2-linoleoylglycerol, and 5-hydroxymethylfurfural [9,18,19]. Pyruvate has anti-inflammatory activity as well as antioxidant activity [47,48]. Pyruvate reduces lipopolysaccharide (LPS)-induced nitric oxide (NO), and prostaglandin E_2_ (PGE_2_) is released [9]. The 2-linoleoylglycerol isolated from ABG suppresses the levels of NO, PGE_2_, and pro-inflammatory cytokines via inhibition of mitogen activated protein kinases signaling pathways in LPS-induced RAW264.7 cells [19]. The 5-hydroxymethylfurfural reduces TNF-α-induced monocytic cell adhesion to human umbilical vein endothelial cells (HUVECs) through suppression of vascular cell adhesion molecule-1 (VCAM-1) expression, ROS generation, and nuclear factor kappa B (NF-κB) activation [18].

ABG decreases the production of NO and pro-inflammatory cytokines in LPS-induced RAW264.7 cells and in septicemic mice [56], TNF-α-induced NF-κB activation in HUVECs [12], and phorbol 12-myristate-13-acetate-induced production in COX-2 and PGE_2_ through inactivation of NF-κB [57]. Hexane extract of ABG reduces cell proliferation and expression of ICAM-1 and VCAM-1 in TNF-α-activated human endometrial stromal cells [20]. *Lactobacillus rhamnosus* fermented ABG inhibits generation of NO, PGE_2_, COX-2, and pro-inflammatory cytokines through upregulation of heme oxygenase-1 in RAW264.7 cells [58]. ABG exerts an anti-inflammatory effect by inhibiting the COX-2 and 5-lipooxygenase activities, pro-inflammatory cytokines, and leukotrienes in LPS-induced RAW264.7 cells [9]. The anti-inflammatory effect of ABG is lower than that of FRG (see Table 4). Generally, anti-inflammatory activity is directly proportional to antioxidant activity, but in ABG it is not [9].

### 3.4. Other Biological Effects

In addition to the above-mentioned antioxidant and anti-inflammatory effects on cells and animals, ABG has anti-cancer, anti-diabetic and obesity, anti-allergic, hepatoprotective, cardioprotective, neuroprotective, and anti-thrombotic effects. The therapeutic effects of ABG have been demonstrated in many types of cells and animal models.

ABG shows dose-dependent chemopreventive effect in several cancers in vitro and in vivo. ABG inhibits cell proliferation and induces apoptosis in SGC-7901 human gastric cancer cells. In Kunming mice inoculated with murine fore-gastric carcinoma cell lines, ABG inhibits growth of inoculated tumors [59]. ABG reduces cell motility, invasiveness, and activities of matrix metalloproteinase-2 (MMP-2) and MMP-9 in AGS human gastric cancer cells [60]. The hexane extract of ABG induces caspase-dependent apoptosis through both intrinsic and extrinsic pathways in U937 leukemic cells [61]. ABG inhibits HT29 colon cancer cell growth via the phosphatidylinositol 3-kinase/protein kinase B (PI3K/Akt) signaling pathway [62]. The 70% and 90% ethanol extracts of ABG have cytotoxicity in several human cancer cell lines: AGS, A549 lung cancer, HepG2 liver cancer, and MCF-7 breast cancer cells [63].

ABG also shows protective effects on diabetes and obese animals. Administration of yeast (*Saccharomyces cerevisiae*) fermented ABG attenuated HFD-increased body fat and plasma lipids in diabetic obese mice [64]. ABG reduces insulin resistance and serum total cholesterol and triglyceride levels, and increases the high density lipoprotein (HDL) cholesterol levels in db/db mice through antioxidant activity [21]. The SAC enriched black garlic juice gives anti-diabetic effects in streptozotocin-induced insulin deficient mice [65]. ABG reduces thiobarbituric acid reactive substances (TBARS) in serum, liver, and kidneys through increasing activities of antioxidant enzyme in streptozotocin-induced diabetic rats [66]. ABG supplementation for 12 weeks reduces blood lipid parameters in patients with mild hypercholesterolemia [67]. In vitro, ABG inhibits adipocyte differentiation and adipogenesis by suppressing the pro-adipogenic transcription factors in 3T3-L1 preadipocytes [68].

ABG has anti-allergic activities inhibiting β-hexosaminidase release in RBL-2H3 rat basophilic leukemia cells [39]. It is also shown to inhibit immunoglobulin E mediated allergic response in RBL-2H3 cells as well as in vivo passive cutaneous anaphylaxis [69].

ABG has protective effects from liver, heart, and brain damage. ABG shows hepatoprotective effect on carbon tetrachloride- or d-galactosamine-induced liver damage and HFD-induced hepatic steatosis and subsequent liver injury in rat [26]. ABG extract enriched with SAC and polyphenols (ABG10+) exerts cardioprotective effects. In rat hearts, ABG10+ induces a relaxing effect on coronary arteries before and after ischemia reperfusion (IR) and prevents the IR-induced decrease in myocardial contractility [70]. ABG ethanol extract increases spatial memory and the number of Purkinje cells in rats treated with monosodium glutamate [71,72], indicating that ABG might have a neuroprotective effect. ABG shows anti-thrombotic effects on thrombin-induced platelet aggregation in rat [73] and human [38]. ABG downregulates heat shock proteins 70 and COX-2 expression levels [74], and reduces TBARS in aerobic exercise rats [74,75]. These various therapeutic effects result from antioxidant and/or anti-inflammatory activities of ABG as shown in aerobic exercise rats. Some hepatoprotective and anti-obesity effects are described in Section 3.2 as anti-oxidative activity.

## 4. Adverse Effects and General Limitations

Compared to FRG, anti-inflammatory [9], anti-coagulation [38], anti-allergic [39], and immunomodulatory [76] effects of ABG are low. Increased sugar concentrations and decreased allicin concentrations during ABG processing cause low anti-inflammatory effects in LPS-activated RAW264.7 cells [9]. Allicin and DADS activate transient receptor potential (TRP) channel [77,78], which is related to a variety of physiological functions. ABG does not affect TRP channels due to low or no allicin level. These different properties may make a significant difference in physiological functions in a variety of cells and tissues. One case study shows that black garlic-induced pneumonia in a 77-year-old female [79]. A drug-induced lymphocyte stimulation test for ABG showed positive results, suggesting a delayed type of immunoreaction rather than cytotoxic reaction. In addition, administration of ABG for a long time could induce problems in the control of bleeding during a surgery, because ABG has anti-coagulation effects.

In addition to its therapeutic effects mentioned in this review, there are likely to be more effects, as FRG or aged garlic do. Regarding cardiovascular functions of garlic, for example, numerous studies have demonstrated that FRG and aged garlic have cardiovascular protective effects on many types of cardiovascular disorders [80,81,82,83,84,85,86,87,88]. However, only one paper reporting that ABG exerts cardioprotective effects on heart during ischemia reperfusion is cited in this review [70]. AGE is effective in reducing blood pressure in uncontrolled hypertensive patients [81] and spontaneously hypertensive rats [82]. In addition, it reduces cardiovascular risk factors in metabolic syndrome rats, and in patients with uncontrolled hypertension [81,83]. AGE attenuates ischemic cerebral damage and myocardial toxicity in rats [84,85,86,87], and exerts endothelium-dependent vasorelaxant effects on rat aorta [88]. More studies are needed to determine the therapeutic effects of ABG.

Components in ABG have not yet been fully analyzed, as there are not enough studies available regarding them. Components and contents vary with aging time and temperature. Moreover, variation of physicochemical properties and contents could result from different garlic cultivars, including agronomic, genetic, and environmental (different culture area) factors [89,90,91,92]. Established manufacturing protocol is needed to analyze the components and contents in ABG and to produce ABG with strong health benefits. Some researchers studying ABG may report their results as aged garlic. In this case, the results would not have been included in our review, because we summarized literature published as ABG and black garlic. Many in vitro and in vivo studies have demonstrated that ABG has a variety of therapeutic effects, including anti-cancer, anti-diabetic, anti-obesity, immunomodulatory, cardioprotective, hepatoprotective, and neuroprotective effects through antioxidant and anti-inflammatory activities. However, additional studies are required to understand biological functions and compositions of ABG in more detail. Understanding biological function, composition, and therapeutic effects could help preventing adverse effects from long-term administration of ABG, and developing health promoting properties.

## 5. Conclusions

Physicochemical properties of FRG change during aging. The aging process increases concentration of flavonoids, pyruvate, total phenol, SAC, free sugars, and minerals. ABG has a sweet and sour taste and jelly-like texture, with dark brown color, and higher concentrations of phytochemical components and free sugars and minerals than those in FRG. ABG has strong antioxidant effects, but low anti-inflammatory, anti-coagulation, anti-allergic, and immunomodulatory activities compared to FRG. These differences between FRG and ABG could result from the concentration of components changing during ABG processing. These different properties should be considered in the choosing of functional foods for their health benefits.

## Figures and Tables

**Table 1:** Comparison of contents of physicochemical components between aged black garlic (ABG) and fresh raw garlic (FRG).

Components	Content in ABG	Content in FRG	Change in ABG Compared to FRG	Analytical Methods	References	Remarks
Moisture (%)	58.2 ± 0.4	66.6 ± 1.3	↓	AOAC 925.10	[31]	
	45.3	66.1	↓		[34]	
	29.9 ± 0.5	64.2 ± 1.5	↓		[37]	
	43.1 ± 0.4	62.3 ± 0.6	↓	AOAC 991.02	[25]	
	45.1	60.3	↓	-	[14]	
Protein (%)	1.0 ± 0.1	0.7	↑	AOAC 984.13	[31]	
	9.1	8.4	↑	-	[14]	
Lipid (%)	0.6 ± 0.1	0.2	↑	AOAC 920.39	[31]	
	0.3	0.1	↑	-	[14]	
Carbohydrate (%)	47	28.7	↑	-	[14]	
Ash (%)	1.8 ± 0.1	0.9 ± 0.6	-	AOAC 942.05	[31]	
	0.11	0.07	↑		[25]	
	2.1	-	↑	-	[14]	no detection in FRG
Total sugar (%)	6.2	4.5 ± 0.1	↑	colorimetric method using PSA	[31]	
	49.2 ± 0.2	14.6 ± 1.3	↑		[38]	
Reducing sugar (%)	1.6	0.2	↑	colorimetric method using DNS	[37]	
	28.2 ± 1.2	4.2 ± 0.1	↑		[38]	
pH	4.4 ± 0.1	6.8	↓	by pH meter	[31]	
	3.7 ± 0.1	6.3 ± 0.1	↓		[37]	
	3.1	6.4	↓		[34]	
	4.2 ± 0.1	6.3 ± 0.1	↓		[25]	
Color Brightness	22.5 ± 0.2	78.8 ± 0.1	↓	Spectrocolori-meter color comparison	[31]	
	4.3 ± 2	68.4 ± 1.7	↓		[37]	
Redness	2.9 ± 0.7	-3.2	↑		[31]	
	2.7 ± 1.0	−3.8 ± 0.5	↑		[37]	
Yellowness	3.2 ± 0.7	21.6 ± 1.4	↓		[31]	
	−3.9 ± 1.5	26.6 ± 1.8	↓		[37]	
Energy (kcal/100 g)	227.1	138	↑	-	[14]	

Brightness ranges from black (0) to white (100). Redness ranges from −60 (green) to 60 (red). Yellowness ranges from −60 (blue) to 60 (yellow). The up-arrow symbol (↑) and down arrow symbol (↓) represent an increase and a decrease in physicochemical components during the aging processes, respectively. AOAC represents Association of Official Analytical Chemists. Comparison between ABG and FRG was performed within a single study.

**Table 2:** Comparison of contents of phytochemical components between ABG and FRG.

Components	Content in ABG	Content in FRG	Change in ABG Compared to FRG	Analytical Methods	Basis for Comparison	References
Allicin (mg/100 g)	-	362 ± 1	↓	colorimetric method	allicin	[9]
	20	345	↓	HPLC		[35]
Flavonoid (mg/100 g)	0.8	0.1	↑	colorimetric method	quercetin	[31]
	1570 ± 211	322 ± 7	↑		rutin	[37]
	195 ± 8	125 ± 13	↑		rutin	[38]
Pyruvate (mmol/100 g)	27.8 ± 0.3	18.8 ± 0.3	↑	Colorimetric method		[31]
	245.7 ± 2.4	48.7 ± 1.2	↑			[9]
Thiosulfate (mmol/100 g)	9.12 ± 0.05	0.65 ± 0.03	↑	colorimetric method		[9]
	0.3	10.5 ± 0.4	↓			[25]
	0.8	0.1	↑		OD value	[31]
Total phenol (mg/100 g)	1.6 ± 0.1	0.6 ± 0.1	↑	Colorimetric method	caffeic acid	[31]
	4835 ± 114	1391 ± 162	↑		garlic acid	[37]
	1000 ± 100	367 ± 22	↑		garlic acid	[16]
	22.3 ± 0.8	3.7 ± 0.2	↑		garlic acid	[39]
	1023 ± 19	255 ± 12	↑		tannic acid	[38]
SAC (mg/100 g)	8.5 ± 0.1	2	↑	HPLC-FLD		[34]
	19.4	2.4	↑	HPLC		[14]
	9.8 ± 0.2	2.2	↑	HPLC-FLD		[15]
	11.4 ± 0.9	2.3	↑	HPLC		[15]

The up-arrow symbol (↑) and down arrow symbol (↓) represent an increase and a decrease in phytochemical components during the aging processes, respectively. FLD represents fluorescence detection. Comparison between ABG and FRG was performed within a single study.

**Table 3:** Comparison of contents of free sugars and minerals between ABG and FRG.

Components		Content in ABG	Content in FRG	Change in ABG Compared to FRG	References	Remarks
Free sugars (mg/100 g)	Arabinose	1.6 ± 0.3	-	↑	[31]	no detection in FRG
		114.5 ± 15.6	51.1 ± 6	↑	[25]	
	Galactose	13.1 ± 1.7	-	↑	[31]	no detection in FRG
	Glucose	181.7 ± 8.8	91.6 ± 2.7	↑	[31]	
		210 ± 5	-	↑	[9]	no detection in FRG
		221.9 ± 11.5	16.7 ± 0.7	↑	[25]	
	Fructose	2043.7 ± 5	63.9 ± 3.4	↑	[31]	
		4002 ± 71	707 ± 8	↑	[9]	
		3383.2 ± 44.0	31.4 ± 1.0	↑	[25]	
	Sucrose	119.1 ± 3.5	76.3 ± 0.1	↑	[31]	
		242.9 ± 18.1	181.7 ± 1.6	↑	[25]	
	Maltose	7.8 ± 0.4	1.7	↑	[31]	
		48.2 ± 4.4	11.7 ± 0.8	↑	[25]	
Minerals (mg/100 g)	Al	25.4 ± 0.8	0.5	↑	[31]	
	Ca	13.1 ± 0.4	7.5	↑	[31]	
		15.2 ± 0.4	12.2 ± 0.3	↑	[25]	
	Cu	2 ± 0.1	1.7	↑	[31]	
		0.27	0.32	↑	[25]	
	Fe	4.4 ± 0.2	3.3 ± 0.2	↑	[31]	
		2.3 ± 0.5	1.6	↑	[25]	
	K	738.2 ± 11.2	434.9 ± 4.2	↑	[31]	
		1018.4 ± 23.2	907.4 ± 20.6	↑	[25]	
	Mg	27.2 ± 0.8	15.7 ± 0.1	↑	[31]	
		48.7 ± 1.1	43.2 ± 1.0	↑	[25]	
	Mn	0.7	0.5	↑	[31]	
		0.5	0.4	↑	[25]	
	Na	13.0 ± 0.1	9.1	↑	[31]	
		35.5 ± 0.8	22.0 ± 0.5	↑	[25]	
	Zn	1.6 ± 0.1	1.5	-	[31]	
		1.3	1.2	↑	[25]	
	P	143.7 ± 5.5	93.3 ± 0.3	↑	[31]	
		2.6 ± 0.1	2.2 ± 0.1	↑	[25]	
	Se	0.13	0.05	↑	[25]	
	S	189.7 ± 4.3	183.1 ± 4.2	-	[25]	

The up-arrow (↑), down arrow (↓), and dash (-) represent an increase, a decrease, and no change in free sugars and minerals during the aging processes, respectively. Comparison between ABG and FRG was performed within a single study.

**Table 4:** Comparison of antioxidant and anti-inflammatory activities between ABG and FRG.

Biological Activities	Measurement	Content in ABG	Content in FRG	Change in ABG Compared to FRG	Basis for Comparison	References
Antioxidant activity	DPPH radical scavenging activity	0.1 mg/mL	0.4 mg/mL	↑	IC_50_	[9]
		4.1 mg/mL	114.9 mg/mL	↑	IC_50_ (Japanese garlic)	[14]
		7.3 mg/mL	88.5 mg/mL	↑	IC_50_ (Chinese garlic)	[14]
		97%	10.4%	↑	900 μg/mL	[16]
		67.4 ± 0.2%	35.7 ± 0.6%	↑	1 mg/mL	[36]
		1.3 mg/mL	0.7 mg/mL	↓	IC_50_	[38]
		82.5 ± 0.5%	35.1 ± 0.7%	↑	2 mg/mL	[39]
	ABTS radical scavenging activity	0.2 mg/mL	0.3 mg/mL	↑	IC_50_	[9]
		1 mg/mL	1.1 mg/mL	↑	IC_50_	[38]
	Hydroxy radical scavenging activity	75 ± 0.7%	60.7 ± 0.2%	↑	2 mg/mL	[39]
	Nitrite radical scavenging activity	32.9%	55.2%	↓	5 mg/mL	[16]
		0.1 mg/mL	0.2 mg/mL	↑	IC_50_	[38]
	Fe^2+^-chelating activity	18.2 ± 0.7%	30.6 ± 1.4%	↓	2 mg/mL	[39]
	Reducing power	2.6	0.4	↑	OD	[39]
	SOD activity	64.4%	16.8%	↑	100 mg/mL	[16]
Anti-inflammatory activity	Inhibition of COX-2 activity	39.1 ± 3.8%	80.5 ± 7.8%	↓	250 μg/mL	[9]
	Inhibition of 5-LO activity	29.5 ± 2.1%	97.4 ± 9.5%	↓	250 μg/mL	[9]

DPPH: 2,2-diphenyl-1-picrylhydrazyl; ABTS: 2,2-azinobis-(3-ethylbenzothiazoline-6-sulfonate); IC_50_: the concentration of a sample that gives half inhibition. The up-arrow symbol (↑) and down arrow symbol (↓) represent an increase and a decrease in antioxidant and anti-inflammatory activities during the aging processes, respectively. Comparison between ABG and FRG was performed within a single study.
